# From prospective biobanking to precision medicine: BIO-RAIDs – an EU study protocol in cervical cancer

**DOI:** 10.1186/s12885-015-1801-0

**Published:** 2015-11-04

**Authors:** Charlotte Ngo, Sanne Samuels, Ksenia Bagrintseva, Andrea Slocker, Philippe Hupé, Gemma Kenter, Marina Popovic, Nina Samet, Patricia Tresca, Heiko von der Leyen, Eric Deutsch, Roman Rouzier, Lisa Belin, Maud Kamal, Suzy Scholl, RAIDs consortium http://www.raids-fp7.eu/

**Affiliations:** 1 Department of Medical Oncology, Institut Curie, 25 Rue d’Ulm, Paris, 75005 France; 2 Department of Gynecology, Netherlands Cancer Institute – Antoni van Leeuwenhoek (NKI-AVL), P.O. Box 90203, 1006 BE Amsterdam, The Netherlands; 3 Department of Radiation Oncology, Institut Gustave Roussy (IGR), 114 Rue Edouard-Vaillant, 94805 Villejuif Cedex, France; 4 INSERM U900, Paris, France; 5 Mines ParisTech, Fontainebleau, France; 6 CNRS UMR 144, Paris, France; 7 Department of Gynecology, Institut of Oncology of Vojvodina (IOV), Put Doktora Goldmana 4, 21204 Sremska Kamenica, Serbia; 8 Department of Radiology Gynecology, Institute of Oncology of Republic of Moldova, str. N. Testemiţanu 30, MD-2025 Chişinău, Republica Moldova; 9 Hannover Clinical Trial Center (HCTC) GmbH, Carl-Neuberg-Str.1, 30625 Hannover, Germany; 10 Institut Curie, 26 rue d’Ulm 75248, Paris, Cedex 05 France; 11 Present address: Department of gynecological and breast oncological surgery, Hôpital Européen Georges Pompidou, 20 rue Leblanc, 75015 Paris, France

**Keywords:** Prospective European biobanking study, Precision medicine, Molecular profiling, Cervical cancer, Next generation sequencing, Reverse phase protein array, Tumor micro environment, Targeted therapy, Patient stratification, International trial, Clinical trial operations, Quality control of pelvic radiotherapy, Biobanking, HPV typing

## Abstract

**Background:**

Cervical cancer (CC) is -second to breast cancer- a dominant cause of gynecological cancer-related deaths worldwide. CC tumor biopsies and blood samples are of easy access and vital for the development of future precision medicine strategies.

**Design:**

BIO-RAIDs is a prospective multicenter European study, presently recruiting patients in 6 EU countries. Tumor and liquid biopsies from patients with previously non-treated cervical cancer (stages IB2-IV) are collected at defined time points. Patients receive standard primary treatment according to the stage of their disease. 700 patients are planned to be enrolled. The main objectives are the discovery of -dominant molecular alterations, -signalling pathway activation, and -tumor micro-environment patterns that may predict response or resistance to treatment. An exhaustive molecular analysis is performed using 1° Next generation sequencing, 2° Reverse phase protein arrays and 3° Immuno-histochemistry.

**Discussion:**

The clinical study BIO-RAIDs is activated in all planned countries, 170 patients have been recruited till now. This study will make an important contribution towards precision medicine treatments in cervical cancer. The results will support the development of clinical practice guidelines for cervical cancer patients to improve their prognosis and their quality of life.

**Trial registration:**

Clinicaltrials.gov: NCT02428842, registered 10 February 2015.

## Background

Cervical cancer (CC) is the second cause of gynecological cancer-related deaths worldwide [1]. Incidence and mortality rates of CC are up to twelve times higher in Eastern Europe as compared to North/Western Europe due to previously inadequate or absent screening practices [1, 2].

HPV is commonly accepted as the major etiological cause of CC [3]. Preventive vaccination is expected to impact incidence rates in more than 20 years from now when the first vaccinated adolescents will reach the age of peak incidence (35–50) of CC. In the meantime, women are at risk and there is an unmet medical need to improve the diagnosis and treatment of CC.

### Diagnosis, standard treatment and prognosis of CC

FIGO (International Federation of Gynecology and Obstetrics) classification for CC was revised in 2009. The use of MRI imaging was encouraged towards improving staging accuracy (92 %) [4–7]. While screening has decreased the incidence of CC in Western EU countries, its treatment has not changed over the last 15 years, the most recent improvement being the association of radio-chemotherapy [8, 9]. Addition of Bevacizumab [10] or Gemcitabine [11] to chemotherapy did show small improvements, but these treatments are costly, have side effects and are not available in many countries in need.

In early stage disease (≤IB1) and in a subset of patients of stage IB2-IIA, a surgical approach may cure the patient [12–15]. For stages IB2 to III, concurrent chemo-radiation with a platinum-based reagent is the recommended standard of therapy [9]. Unfortunately, advanced stage (III-IV) disease remains a significant public health problem [16]. In on-going clinical trials, the impact of additional chemotherapy either as neo-adjuvant (INTERLACE trial [17] or adjuvant (OUTBACK trial: ASCO meeting 2012) strategies, are currently being assessed.

### Precision medicine in CC and BIO-RAIDs objectives

Molecularly targeted agents in CC are still being tested in clinical trials. CC patients may have variable outcomes and specific genetic mutations have already been shown to impact clinical response to standard therapy by us [18] and others [19–21]. The Cetuxicol trial, a phase 2 trial sponsored by Institut Curie, showed that the addition of Cetuximab over a 6 week period, did not improve DFS. Of interest was the finding that PI3K pathway tumor mutations in the Cetuximab treatment arm led to a worse outcome (De la Rochefordiere 2015). We are presently lacking prognostic and predictive biomarkers for CC treatment and there is a growing need for the development of these to follow up the course of the disease. Due to the multiplicity of potential genetic alterations, retrospective molecular assessments in small patient populations are mostly inconclusive. For these reasons, we initiated BIO-RAIDs, a prospective study with extensive biobanking which aims to identify predictive biomarkers for treatment response of cervical cancers in both Western and Eastern European countries. To our knowledge, BIO-RAIDs is the first large prospective trial of this type in the field of CC. At the medical/scientific level, BIO-RAIDs will be crucial in setting the ground for future precision medicine studies by identifying a set of stratification criteria for cervical carcinomas as well as other cancers with similar molecular alterations.

## Methods

**Fig. 1:**
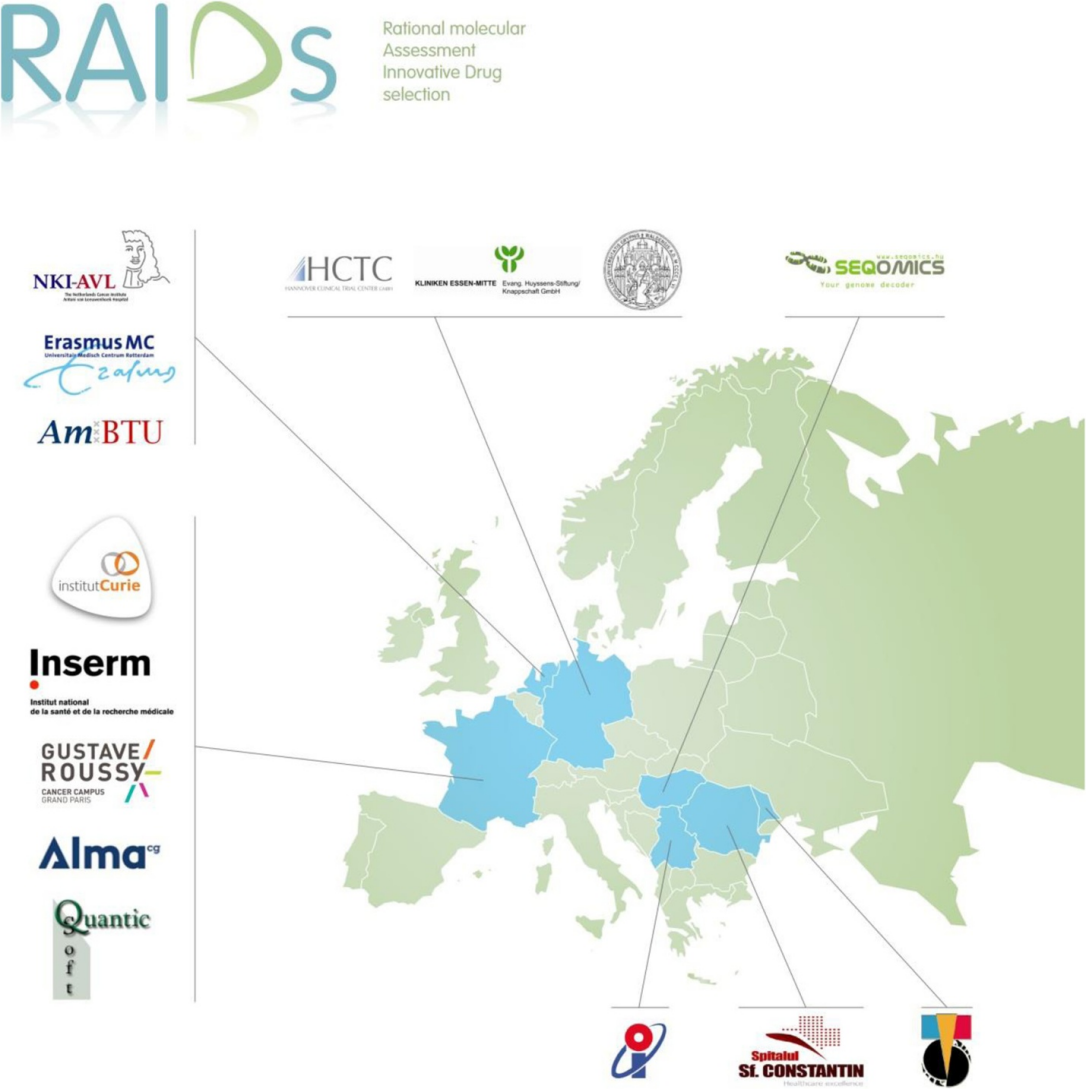
RAIDs project - participant countries. RAIDs is an international multidisciplinary cooperation between academic hospitals, SMEs and platforms for translational research (http://www.raids-fp7.eu/). 7 EU countries participate in RAIDs project: France, Hungary, Germany, Netherlands, Serbia, Moldova and Romania. BIO-RAIDs study is performed in 6 EU countries: France, Germany, Netherlands, Serbia, Moldova and Romania

The BIO-RAIDs study is at the core of the European project called RAIDs “Rational molecular Assessment Innovative Drug selection” coordinated by Institut Curie. RAIDs is based on an international multidisciplinary cooperation between academic hospitals, SMEs and platforms for translational research www.raids-fp7.eu/ (Fig. 1).

Institut Curie (France) is responsible for the overall coordination and management (study documents and data quality, statistical analyses). In countries other than France, the registration, management, and monitoring of clinical centers is delegated to a national coordinator.

### Study design and objectives

**Fig. 2:**
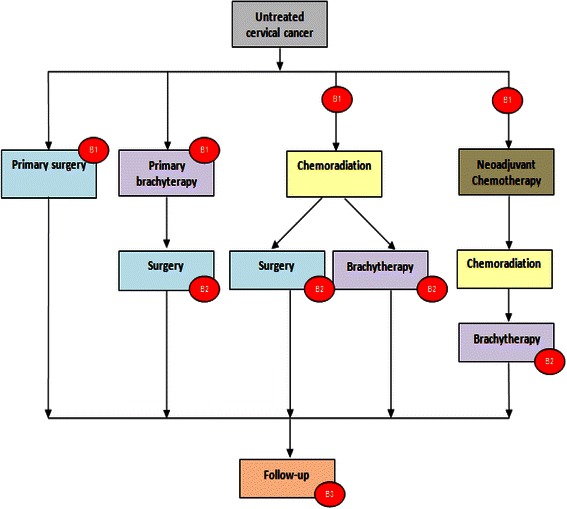
Recommended treatment and time of biopsies. Patients with previously untreated, advanced stage cervical cancer (stage IB2-IV) will be enrolled in BIO-RAIDs study and undergo standard treatment. The recommendations for standards of treatment by stage and the timing of biopsies are shown. B1: biopsy before treatment; B2: biopsy during or at the end of treatment in case of poor response/progression; B3: biopsy in case of recurrence

BIO-RAIDs is a prospective European study, involving oncology centers from France, Germany, Serbia, the Netherlands, Romania and Moldova. 700 patients with previously untreated, advanced stage CC (stage IB2-IV) will be enrolled. The recommendations for standards of treatment by stage of disease as well as the timing of biopsies are shown in Fig. 2. The primary objective of the study is to assess dominant mutations and activation of signaling pathways in cervical cancers predictive of response to standard treatment. Secondary objectives of the study are: 1. The determination of PFS at 18 months as a function of dominant genetic or protein alterations; 2. The descriptive analysis of standard treatments applied in participating European countries; 3. A descriptive analysis of grade 3 and 4 side effects; 4. A descriptive analysis of the frequency of molecular alterations according to geographic location.

### Ethical considerations and regulatory approvals

Approval for the BIO-RAIDs study has been obtained from ethics committees in all participating countries (Comité pour la protection des personnes (CPP) Ile de France in France, The Serbian Medical Society Belgrade in Serbia, The Protocol Toetsingscommissie of the Antoni van Leeuwenhoek (PTC) in the Netherlands, The Medizinische Hochschule Hannover Ethik-kommission in Germany, The National Ethics Committee of Health Ministry of Republic of Moldova, Academy of Medical Sciences National Ethics Committee for medicines and medical devices in Romania). BIO-RAIDs is conducted in accordance with the guidelines of the Declaration of Helsinki, and the principles of Good Clinical Practice as defined by the International Conference on Harmonization (ICH-E6, 17/07/96) as well as specific laws and regulations of the countries were the study is performed. Centers participating in BIO-RAIDs study are listed in the Table 1.

**Table 1:** Centers participating in the BIO-RAIDs study. All the participating centers in the BIO-RAIDs study on the moment of submission are shown

France	Institut Curie - Paris, Institut Bergonie - Bordeaux, Institut Rene Huguenin Curie - Saint Cloud, Institut Gustave Roussy – Villejuif, Institut de Cancérologie Lorraine – Nancy, Centre Georges François Leclerc – Dijon ; Institut de Cancérologie de l'Ouest - Nantes ; Institut de Cancérologie de l'Ouest - Paul Papin – Angers ; Hôpital Européen Georges Pompidou - Paris; Institut régional du Cancer Montpellier (Val d'Aurelle) – Montpellier and Centre Antoine Lacassagne-Nice.
Germany	Hannover Medical School – Hannover, Carl-Gustav Carus University - Dresden
The Netherlands	Netherlands Cancer Institute – Antoni van Leeuwenhoek (NKI-AVL) - Amsterdam, Amsterdam Medical Center - Amsterdam
Romania	Clinica Radiotherapie Timisoara, Spitalul Clinic Municipal – Oradea, Institutul Regional de Oncologie - Iasi
Serbia	Institut of Oncology of Vojvodina (IOV) – Novi Sad
Moldova	Institute of Oncology of Republic of Moldova - Chisinau

### Patient recruitment, data collection and biobanking

Eligible patients with stage IB2-IV disease, scheduled for primary surgery, chemo-radiation or primary chemotherapy are invited to participate in this study. Table 2 details the inclusion and exclusion criteria. Documented informed consent is obtained for all patients. Patient data is anonymized and recorded in an electronic Case Report Form (eCRF) (Quanticsoft). The eCRF generates patient specific unique barcode numbers for each sample. At study entry, baseline demographic characteristics, medical history and findings of staging such as complete physical and gynecological examination, abdominal and pelvic CT, pelvic MRI (+/- optional PET-CT) are recorded. A central review of MRI imaging is planned to be performed in Serbia.

**Table 2:** Inclusion and exclusion criteria

Inclusion criteria	Exclusion criteria
1) No prior treatment for CC.	1) Patient enrolled in a clinical trial involving an investigative new agent.
2) FIGO Stage IB2 to IVB; all histological subtypes (excluding neuro-endocrine type).	
	2) Co morbidity, preventing patient to tolerate the proposed standard treatment.
	3) Past history of invasive cancer over the 5 years preceding entry in the present trial (except basal cell carcinoma and carcinoma in situ of the cervix).
3) Pelvic MRI available or planned before the start of treatment.	
4) Possibility to communicate imaging data by CD ROM (format DICOM 3.0 or more).	4) Impossibility to carry out evaluation by MRI (patient claustrophobic, pacemaker, metallic implant, non-availability, other).
	5) Patient deprived from ability to decide on her own.
5) Disease amenable to biopsy (3 tumor samples are mandatory prior to treatment).	
	6) Patient unable to have a regular follow up for geographical, social or psychological reasons.
6) Age ≥ 18 years.	
	7) Pregnancy or patient old enough to procreate and not using effective contraceptive method.
2) ECOG 0-2.	
3) Life expectancy > 6 months.	
4) Patient eligible for standard treatment (according to standards of each center).	
5) Patient having health care insurance.	
6) Informed and signed consent by patient.	

### Biobanking: sample collection and procedures

Tumor and serum samples are collected at defined time points as shown in Table 3 and stored at −80 °C (mutational data) or at room T° in case of preparation of fixed paraffin embedded sections (IHC). Standard operating procedures have been established by the RAIDs consortium for biopsy handling as well as for blood and sera collections. All samples are centralized in the RAIDs biobank located in Morahollum (SeQomics, Hungary).

**Table 3:**
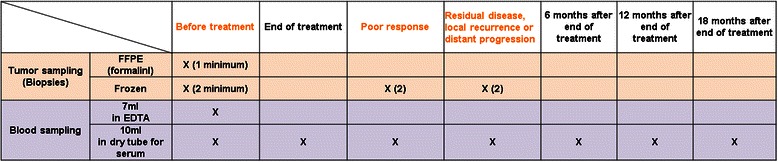
Biobanking during BIO-RAIDs study

#### Tumor samples

One biopsy composed of at least 3 tumor samples must be collected at baseline before any treatment. In the case of large cervical lesions, 2 additional tumor samples may be collected: 1 tumor sample will be paraffin-embedded (FFPE) for Immunohistochemistry analysis and 2 to 4 tumor samples will be instantly frozen in liquid nitrogen and stored at −80 °C. The minimum size for tumor sample is 0.3 cm3 (bite of 7*3*1.5 mm). The ideal size is however > 0.3 cm3, and preferably 0.5–1 cm3. In case of poor response during the primary treatment sequence (at the insertion of the brachytherapy device which is done under general anesthesia following radio chemotherapy or at in case of surgery), 2 additional tumor samples may be collected and instantly frozen in liquid nitrogen and stored at −80 °C. At the end of the patient’s primary treatment sequence and in case of accessible residual/recurrent disease, 2 residual tumor samples will be collected and instantly frozen in liquid nitrogen and stored at −80 °C.

#### Blood sampling

Before treatment, 7 mL of blood is collected in EDTA tubes for DNA extraction and sequencing at Seqomics and 10 mL of blood collected in adapted tubes for serum preparation. At the end of treatment and during follow-up visits at 6 and 12 months 10 mL of blood will be collected in adapted tubes for serum preparation. Sera will be stored at −80 °C and centralized at Seqomics.

#### Sample labelling

Each sample (tumor, blood or serum) is meticulously labeled with a unique 2D barcode provided by Institut Curie at the beginning of the study. For each label a patient specific kit number is assigned by the electronic CRF software at the time of patient inclusion.

**Fig. 3:**
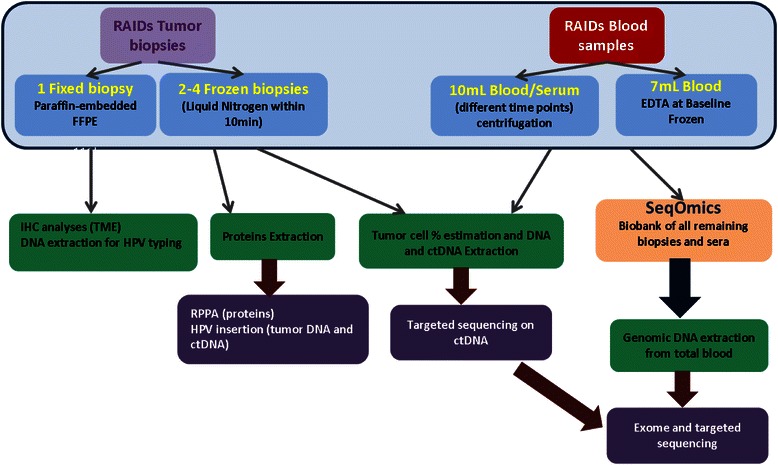
From sample to technique – sample flow. Patient samples (blood samples and biopsies) will be centralized at local centers and then sent to research platforms, where the material will processed and analyzed by different methods ( IHC, HPV insertion, sequencing, RPPA). Centralized biobanking of remaining material will be performed at SeqOmics (Hungary)

**Fig. 4:**
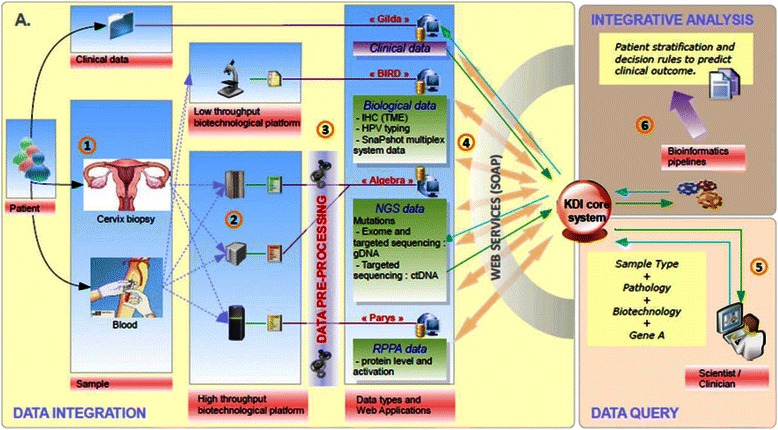
KDI – Knowledge and Data Integration. Integration of heterogeneous clinical and biological/molecular data requires a powerful information system. Data integration: all data (clinical data from eCRF, biological data, including tumor microenvironment (TME) analysis using immunohistochemistry (IHC) and HPV typing and raw data from technological platforms: exome and targeted sequencing on gDNA and ctDNA, reverse phase protein analysis (RPPA)) will be integrated into KDI core system. Afterwards advanced research functionalities will enable multiple data queries. Specific bioinformatics pipelines will generate new integrative knowledge from these heterogeneous sources of data (figure adapted from Servant et al. [22])

Molecular profiling is performed as summarized in Fig. 3. Raw data of molecular profiling together with the clinical data of patients are integrated into a common repository (KDI: Knowledge and Data Integration) (Fig. 4), developed by Institut Curie and already successfully used for the European project MAARS - (261366) as well as for the SHIVA clinical trial at Institut Curie [22].

### Clinical follow-up

Early clinical follow up may vary according to the chosen treatment strategy and a function of the FIGO stage of the disease. Detailed clinical and imaging evaluations are carried out at the end of treatment and at 6, 12 and 18 months. All clinical, sampling and imaging data are registered in the eCRF.

### Quality control for radiotherapy

Standardization for External Beam Radio-chemotherapy (EBRT) and Brachytherapy (BT) delineation in participating centers was improved through online workshops (ODW) according to the GEC-ESTRO recommendations [23] and the EMBRACE protocol (An International study on MRI-guided Brachytherapy in locally advanced CC: EMBRACE. https://www.embracestudy.dk/). Centers with deviations from the treatment protocol are being offered an on-site training period in a reference center. Contour evaluation methodology: Intraobserver variability between 3 contouring periods: 1. *Quantitative differences:* DICE index. 2. *Qualitative assessment:* target Volumes, areas contoured. Organs at Risk A: Optimal: > 0.81; B: Suboptimal: < = 0.81 (Breunig et al. IJROB 2012) A: Optimal: > 0.81 Target Vol. B: Average: 0.65 – 0.81 C: Suboptimal: < 0.65 (*Dimopoulos et al. Radioth Oncol*^2009^; *Petersen et al. IJROB*^2007^*)* Initial analyses demonstrated interobserver variability for baseline contouring. Quantitative analyses were performed between centers and years of experience. Qualitative analyses compared group contours with reference contours. ANOVA was applied for analysis based on DICE, the significance of: institution; organ at risk (OAR) versus target volume (TV); organ; participant’s years of experience (grouped in 2 levels: Residents vs. Specialists). As an example of results of contouring workshop: most centers have an average DICE index for each volume between 0.65 and 0.81. This loosely falls within the average (B) category. If the participants improve in the guideline and final contouring sessions, these centers are initially prepared to participate within the RAIDs study, and only would need to complete a dummy run to validate the dosimetry as well. Half of the RAIDs institutions have suboptimal (C) DICE indexes for the *GTV-node volume*. The qualitative analyses showed some conceptual errors: 1. delineating vessels or clinical nodal target volumes instead of the actual macroscopic lymph nodes. 2. these results are partly due to the clinical case itself: 1. paraaortic lymph node which on the computed tomography image set for contouring seemed enlarged, although the clinical information explicitly stated that no paraaortic lymph nodes were pathological. 2. suspicious lymph node in the left groin, which within the live sessions was deemed as inflammatory by an experienced radiotherapist.

Acute or delayed toxicities of treatment will be documented in the eCRF.

### Bioinformatics and statistical analysis

We try to assess which are the dominant mutations and which signaling pathways are activated in cervical cancers based on potentially deleterious genomic or proteomic alterations (COSMIC database). Furthermore we want to ascertain which abnormalities will be predictive of response to standard treatment and outcome. Analysis of a large number of patients is necessary because if a specific driver mutation is present in only 7 % of patients, 700 patients need to be evaluated to detect this mutation in 50 patients. No statistical hypothesis applies, but appropriate statistical methods will be used to analyze the results.

1. *Definitions:*

Complete response (CR) is assessed by MRI at the end of treatment (latest at 6 months) based on RECIST criteria. In the case of surgery, complete response will be defined as a pathological complete response. Progression-free survival (PFS) is defined as the time from diagnosis to the date of the first progression or death. If patients are alive and free of progression, they will be censored and their PFS will be defined as the time from diagnosis to the date of last known follow-up visit. Overall survival (S) will be defined as the time from diagnosis to the date of death or last follow-up. Survival rates will be estimated using the Kaplan-Meier method. PFS and OS will be compared to molecular phenotypes or clinical factors using the log-rank test.

2. *Statistical methodology*

Univariate and multivariate analyses will be performed to evaluate the association between each set of biomarkers (gain or loss of function) and clinical outcome. If the outcome measure is CR, a logistic regression is used. If the outcome measure is PFS or OS, a Cox regression model is preferred using 95 % confidence intervals. Multivariate analyses will take into account correlations between the different factors allowing to define a genomic or proteomic “response signature”, capable to predict the different outcome measures as compared to the presently available criteria. The level of significance is fixed at 5 %. Corrections for multiple testing to correct for the occurrence of false positives will be by the Benjamini-Hochberg method. Stratification according to geographical location will take into account the heterogeneity of standard treatment. Analyses will be performed using R° software by the Biostatistics Department of Institut Curie.

3. *Bioinformatics*

The bioinformatics’ platform at Institut Curie together with SeQomics (Hungary) will ensure reliable downstream bioinformatics analysis of patient samples. The tumors will be characterized by a list of features such as: mutations, structural variants, protein expression +/- phosphorylation, and presence of an immune signature by IHC among others. In an *unsupervised analysis*, an exhaustive exploration of the molecular profiles, without an a priori model will be carried out to characterize the dominant molecular abnormalities. This approach will involve a principal component analysis, an independent component analysis as well as clustering. This exploratory analysis is meant to detect possible biases due to technological aspects or sample handling (batch effect). The *supervised analysis* will attempt to identify and validate biomarkers using machine learning techniques such as LASSO, ridge regression, elastic net or SVM. The prediction of the influence of specific molecular abnormalities on patient outcome needs to be validated by their impact on the major endpoints which have been defined above: 1° Complete Response (CR). 2° Progression-free survival (PFS); 3° Overall survival (OS). Finally, results of both the unsupervised and supervised analyses will be compared to published classifications.

4. *Biomarkers identification*

The biomarkers identified in the second step will be integrated with well-known clinical (FIGO stage, node involvement etc.) and histological prognostic factors in a multivariate model as defined in the first step. Our objectives are to study correlations and prioritize markers for their distinctive ability to predict complete response, progression free survival and overall survival.

## Discussion

BIO-RAIDs is one of the first prospective studies including a substantial biobanking effort for molecular profiling using fresh frozen tumor material with high standards of quality control of both biological samples and clinical data. While the aim of this study is to assess the relevant impact of dominant genetic/proteomic or immune parameters on primary treatment outcome in a prospective well controlled patient population with sufficient numbers to draw valid conclusions, there were a number of shortcomings in the initiation phase of this trial.

The clinical study BIO-RAIDs is now activated in all planned countries -up to two years after the start of the EU project- and patient recruitment numbers are satisfactory. Multiple bottlenecks causing the delay in this international study initiation were identified. Significant delays in the provisional time frame guidelines were caused by 1° Regulatory aspects; 2° Insurance modalities; 3° Negotiation of sponsorship delegation contracts; 4° Site specific logistics for biobanking; 5° Clinical trials operational management. Based on our experience, we believe there is a real need to develop procedures that facilitate the implementation of trials with biobanking in the era of precision medicine.

## Conclusion and perspectives

The present protocol may serve to model the relationship of molecular aberrations to outcome in cervical cancer. Moreover this may apply to other cancers as well, since treatment response and outcome of a variety of cancers does not segregate according to histological tumor type. Response to treatment may in fact be more closely related to molecular driver genes than to tumor histotype. The implication of this project for the clinical practice of the future is to stratify cancer patients for the most appropriate treatment option.

Knowing the relative risk of good or bad outcome of specific tumor deregulations will be instrumental in guiding us towards more specific and less toxic treatments while also allowing the right amount of supervision and treatment, appropriate for each patient. In the RAIDs project, 20 cell lines have been collected and have been analyzed for molecular markers and for treatment response to a large panel of drug combinations. These may serve as companion diagnostics tools in the future.

## Abbreviations

CC: Cervical cancer
COSMIC: Catalogue of somatic mutations in cancer
CR: Complete response
CTCAE: Common terminology criteria for adverse events
ECOG: Eastern cooperative oncology group
eCRF: Electronic case report file
FIGO: International Federation of Gynecology and Obstetrics
GOG: Gynecologic oncology group
HPV: Human papilloma virus
PFS: Progression-free survival
CRO: Clinical research organization
KDI: Knowledge and data integration
MRI: Magnetic resonance imaging
OS: Overall survival
RAIDs: Rational assessment and innovative drug selection
